# Research progress on bacterial outer membrane vesicles in antibiotic resistance and clinical anti-infective therapy

**DOI:** 10.3389/fmicb.2025.1670307

**Published:** 2025-09-04

**Authors:** Yukang Lu, Zhenzhen Wen, Xiaoyan Liu, Tingting Zhang, Meijin Liu, Linghan Zhang, Jinyou Qiu, Maoyuan Wang

**Affiliations:** ^1^People’s Hospital of Ganzhou Economic Development Zone, Ganzhou, China; ^2^Department of Rehabilitation Medicine, The First Affiliated Hospital of Gannan Medical University, GanZhou, China

**Keywords:** bacterial outer membrane vesicles, antibiotic resistance, gram-negativebacteria, clinical anti-infective therapy, vaccine development

## Abstract

In recent years, bacterial outer membrane vesicles (OMVs)—nanoscale, bilayered membrane structures secreted by Gram-negative bacteria—have attracted considerable attention for their involvement in antibiotic resistance and potential in clinical anti-infective strategies. OMVs encapsulate diverse biomolecules, including proteins, lipids, toxins, and nucleic acids, thereby serving as critical mediators of communication between bacteria and host cells. They contribute to horizontal gene transfer, signal transduction, and biofilm formation, ultimately enhancing bacterial adaptability and resistance. Clinically, OMVs are regarded as promising therapeutic platforms owing to their excellent biocompatibility and intrinsic immunogenicity, with ongoing investigations exploring their roles in vaccine development, targeted drug delivery, and immune modulation. This review highlights the participation of OMVs in resistance mechanisms across common pathogenic bacteria and discusses their emerging applications in infection control. By elucidating the biogenesis and functional mechanisms of OMVs, novel antibacterial strategies may be developed, offering new avenues to address the escalating global challenge of antibiotic resistance.

## 1 Introduction

In recent years, the global public health burden caused by bacterial infections has been increasingly severe, with antibiotic resistance emerging as a major challenge faced by countries worldwide. According to the World Health Organization (WHO), bacterial resistance is spreading at an alarming rate, threatening human health and the safety of healthcare systems (Bertagnolio et al., 2024). Approximately 4.71 million people die annually due to infections by resistant bacteria, and this number is projected to reach 8.22 million by 2050 (Collaborators GBDAR, 2024). The emergence of resistant bacteria not only prolongs patient treatment times but also significantly increases healthcare costs. The development of antibiotic resistance is a complex, multifactorial process involving bacterial genetic variation, environmental pressures, and improper use of medications. This issue is particularly exacerbated in low- and middle-income countries due to the overuse and misuse of antibiotics, which accelerates the spread of resistant strains (Luo et al., 2024). Therefore, uncovering new mechanisms of bacterial resistance to provide a theoretical basis for curbing the emergence and spread of multidrug-resistant bacteria has become a key focus in the fields of microbiology and clinical anti-infective research.

In this context, bacterial outer membrane vesicles (OMVs)—nanoscale, bilayered vesicles naturally released by Gram-negative bacteria—have attracted increasing research interest (Xia et al., 2025). OMVs participate not only in core bacterial physiological processes but also in the pathogenicity and antimicrobial resistance of many clinically relevant bacteria (Gao and van der Veen, 2024). They enclose a diverse repertoire of biomolecules, including proteins, lipids, toxins, and nucleic acids, thereby mediating intercellular communication between bacteria and host cells (Liao et al., 2025). OMVs contribute to horizontal gene transfer, signal exchange, and interbacterial competition, shaping bacterial adaptability and survival (Tong et al., 2024; Baryalai et al., 2025). Furthermore, they are integral to biofilm formation and maintenance, factors that substantially enhance bacterial tolerance to antibiotics (Esoda and Kuehn, 2019).

In clinical applications, the unique properties of OMVs make them potential tools for anti-infective therapy. Through in-depth studies of the structure and function of OMVs, researchers are exploring their applications in vaccine development, drug delivery, and immune modulation (Li et al., 2025). As natural nanocarriers, OMVs offer excellent biocompatibility and immunogenicity, providing new avenues for developing novel anti-infective therapies.

Based on this, the present review systematically elucidates the latest research progress on OMVs from two perspectives. First, it delves into the resistance mechanisms mediated by bacterial OMVs in various common pathogenic bacteria. These pathogens frequently cause human infections, and understanding their resistance mechanisms is crucial for addressing the challenge of bacterial resistance. As the Chinese proverb goes, “Know yourself and know your enemy, and you will never be defeated,” a thorough understanding of bacterial resistance mechanisms is essential for devising effective counter-strategies. Second, the review summarizes the application research of bacterial OMVs in clinical anti-infective therapy. This section embodies the strategy of “using the enemy’s spear to attack the enemy’s shield,” by leveraging the inherent properties of bacterial OMVs to develop new antibacterial approaches. Through the summary and analysis of these two sections, this review aims to provide researchers with new perspectives and ideas, with the ultimate goal of fully revealing the survival strategies of bacteria utilizing OMVs and developing novel antibacterial strategies to address the increasingly severe issue of bacterial resistance.

## 2 OMVs

OMVs are nanoscale membrane structures released by Gram-negative bacteria during growth, formed through the budding and shedding of the outer membrane, typically ranging in diameter from 20 to 250 nm (Abolhasani et al., 2025). They originate from the outer membrane via localized budding and subsequent vesicle scission. Structurally, OMVs comprise a phospholipid bilayer derived from the parent bacterial outer membrane, enriched with lipopolysaccharides (LPS), phospholipids, outer membrane proteins, and other bacterial components. This molecular cargo enables OMVs to engage in diverse interactions with host cells and other microorganisms (Li et al., 2024).

Biogenesis of OMVs is a multifactorial process influenced by both intrinsic cellular factors and extrinsic environmental stimuli. It generally involves transient detachment of the outer membrane from the underlying peptidoglycan layer, allowing outward protrusion followed by vesicle release. Perturbations in the molecular linkages stabilizing this interaction are critical. For example, a reduction in covalent binding between Braun’s lipoprotein (Lpp) and peptidoglycan markedly increases vesicle formation, as does the weakening of non-covalent interactions between outer membrane protein A (OmpA) and peptidoglycan (Schwechheimer and Kuehn, 2015). Membrane lipid composition and LPS structural modifications further regulate OMV production; alterations in lipid fluidity or curvature, as well as specific LPS modifications, can promote vesiculation by modulating membrane bending (Juodeikis and Carding, 2022). Environmental stressors—including elevated temperature, oxidative stress, and antibiotic exposure—also stimulate OMV release, potentially as a bacterial defensive mechanism for removing damaged proteins and lipids (Li et al., 2023). These factors act in concert to precisely modulate OMV yield and composition.

Biological functions of OMVs span bacterial physiology, pathogenicity, and ecological adaptation. They serve as potent mediators of bacteria–host interactions, transporting toxins, enzymes, signaling molecules, and other effectors that can modify host cellular activities and immune responses (Ge et al., 2025). For example, research by Folliero et al. (2022) found that OMVs from *Escherichia coli* have a significant negative impact on human sperm function. They demonstrated that OMVs significantly reduce sperm motility and increase the proportion of immotile sperm, with these effects becoming apparent after 45 min of exposure and intensifying over time. Moreover, OMVs significantly elevated reactive oxygen species (ROS) levels and DNA fragmentation in sperm cells, indicating that oxidative stress might be a key mechanism by which OMVs cause sperm damage. In another example, OMVs from *Helicobacter pylori* were found to activate the NF-κB pathway and stimulate interleukin-8 (IL-8) secretion in gastric epithelial cells. While *H. pylori* whole cells induced higher IL-8 expression levels than OMVs, vesicles isolated from patients with different gastric diseases varied in IL-8 induction capacity, suggesting a potential role in disease pathogenesis (Choi et al., 2021).

Beyond host–pathogen interactions, OMVs facilitate inter-bacterial communication by transporting antibiotic resistance genes and antimicrobial peptides, thereby enhancing bacterial survival under adverse conditions (Chen et al., 2023). They also contribute to biofilm formation and maintenance by delivering adhesins and signaling molecules that promote surface attachment and strengthen biofilm integrity (Lee et al., 2025). These functions collectively enhance bacterial tolerance to antimicrobial agents and adaptability to fluctuating environments, conferring a substantial survival advantage in complex microbial ecosystems.

## 3 Methods for isolation and identification of bacterial OMVs

For OMVs to be applied clinically, efficient, rapid, and stable extraction methods that preserve the structural integrity and biological functionality of OMVs are essential (Preto et al., 2025). Current techniques for OMV isolation and purification each have their advantages and disadvantages. Common laboratory methods for OMV isolation include ultracentrifugation, density gradient centrifugation, ultrafiltration, and immunoaffinity chromatography (Table 1). Researchers should choose different isolation methods based on their laboratory conditions and experimental objectives.

**Table 1:** Common methods for isolation and purification of bacterial outer membrane vesicles.

Method	Procedure	Advantages	Disadvantages
Ultracentrifugation	1. Collect bacterial culture supernatant and remove cell debris. 2. Pellet OMVs using ultracentrifugation (typically 100,000 × g, 2–4 h). 3. Resuspend OMVs in suitable buffer and repeat ultracentrifugation for further purification.	Relatively simple; suitable for routine laboratory use; yields high-purity OMVs.	Requires expensive ultracentrifuge; time-consuming; relatively low efficiency.
Density Gradient Centrifugation	1. Place OMV suspension (from ultracentrifugation) onto a density gradient medium (e.g., sucrose or iodixanol). 2. Perform ultracentrifugation to separate OMVs by density. 3. Collect OMV fractions of specific densities.	Further increases OMV purity; allows separation of OMV subpopulations with different densities.	Complex operation; requires precise control of density gradient; more time-consuming and costly.
Ultrafiltration	1. Filter bacterial culture through ultrafiltration membranes (e.g., 100 kDa cutoff) to remove macromolecular impurities. 2. Collect filtrate and further concentrate OMVs.	Equipment is relatively inexpensive; simple operation; suitable for large-scale processing.	Lower OMV purity compared to ultracentrifugation; may require multiple rounds to improve purity.
Immunoaffinity Chromatography	1. Immobilize specific antibodies (e.g., against OMV surface proteins) on a chromatography column. 2. Pass the bacterial culture through the column to capture OMVs. 3. Elute OMVs with elution buffer.	Yields high-purity OMVs; enables selective capture of specific OMV subpopulations.	Requires specific antibodies; expensive; complex operation; requires optimization of antibody immobilization and elution conditions.
Immobilized Metal Affinity Chromatography (IMAC)	1. Introduce a His-tag on the OMV surface. 2. Purify OMVs using Ni-NTA resin. 3. Elute OMVs with elution buffer (e.g., containing high concentrations of imidazole).	Simple operation; relatively low cost; selective purification of His-tagged OMVs.	Requires genetic engineering of OMVs; metal ions may affect OMV bioactivity.
Precipitation	1. Treat bacterial culture with specific precipitants (e.g., polyethylene glycol, ammonium sulfate). 2. Collect precipitated OMVs by centrifugation.	Simple operation; low cost; suitable for large-scale processing.	Lower purification efficiency; often requires further purification; precipitants may affect OMV bioactivity.

Single-step procedures often fail to meet high-purity and high-yield requirements, making combined approaches preferable. For example, Li et al. (2024) developed a modified one-step OptiPrep density gradient ultracentrifugation (DDGC) technique for *Klebsiella pneumoniae* OMVs. Bacterial culture supernatants underwent differential centrifugation to remove cell debris, followed by OptiPrep density gradient centrifugation for OMV separation. Compared with conventional differential centrifugation, the DDGC method produced OMVs of more uniform size, clearer background, characteristic morphology, higher lipopolysaccharide and outer membrane protein content, and enhanced biological activity—manifested as greater cytotoxicity toward A549 lung epithelial cells and increased induction of inflammatory cytokines.

Similarly, Alves et al. (2017) demonstrated a His-tag–based immobilized metal affinity chromatography (IMAC) strategy for rapid and selective OMV purification. By genetically engineering the outer membrane protein A (OmpA) to display an N-terminal His-tag, they enabled efficient capture and elution of *Escherichia coli* OMVs from complex media. This approach is cost-effective, scalable, and compatible with downstream applications in pharmaceuticals, environmental remediation, and basic research.

Similar to the identification of small extracellular vesicles, OMVs can be identified by observing their morphology and size distribution using transmission electron microscopy and scanning electron microscopy. Nanoparticle tracking analysis provides quantitative analysis of OMV concentration and size distribution (Xing et al., 2025). However, unlike small extracellular vesicles, the International Society for Extracellular Vesicles (ISEV) has specified certain marker proteins for the identification of small extracellular vesicles, whereas protein identification for OMVs is more challenging (Welsh et al., 2024). Unlike small extracellular vesicles, OMVs typically contain outer membrane proteins, periplasmic proteins, and some cytoplasmic proteins, with specific types and proportions varying depending on bacterial species and environmental conditions. Therefore, OMV identification usually relies on morphological observation and compositional analysis rather than specific marker proteins.

## 4 Bacterial OMVs mediated antibiotic resistance

### 4.1 Klebsiella pneumoniae

*Klebsiella pneumoniae* is a significant pathogen with increasingly severe antibiotic resistance issues, particularly against key antibiotics such as polymyxins and carbapenems. In recent years, the critical role of OMVs in the resistance mechanisms of *K. pneumoniae* has become clearer. OMVs not only directly protect bacteria from antibiotic attacks but also facilitate the spread and expression of resistance genes through various mechanisms, significantly complicating the resistance problem.

OMVs can directly shield bacteria from antibiotic attacks. Burt et al. (2024) reported that polymyxin B exposure markedly increases OMV secretion by *K. pneumoniae* while reducing vesicle size. These vesicles competitively bind polymyxin B, protecting bacteria *in vitro*, ex vivo, and *in vivo*, thereby enabling growth even in antibiotic-rich environments. Similarly, Lucena et al. (2023) demonstrated that OMV protein profiles of multidrug-resistant *K. pneumoniae* (KpHCD1) are remodeled by sub-inhibitory antibiotic concentrations (meropenem, amikacin, polymyxin B, and combinations). LC–MS/MS identified 64 differentially abundant proteins spanning resistance factors (OmpA, OmpK37, KPC-2 carbapenemase), virulence factors (AcrAB efflux pump, fimbrial proteins, iron uptake receptors), and stress-response proteins (ribosomal proteins, FkpA, Spy). Antibiotic-specific effects included: meropenem enrichment of cell division proteins (NlpD, MltA); polymyxin B upregulation of energy metabolism proteins; and amikacin-induced suppression of OMV production.

Horizontal gene transfer (HGT) refers to the direct exchange of genes between different organisms, rather than vertical gene transfer from parent to offspring (Thomas and Nielsen, 2005). HGT is widespread in the microbial world, especially among bacteria, playing a crucial role in bacterial adaptation to environmental changes, acquisition of resistance, and virulence factors. HGT is a key mechanism for bacteria to develop multidrug resistance, allowing resistance genes to rapidly spread across different species (Frost et al., 2005; Nino-Vega et al., 2025). Dell'Annunziata et al. (2021) studied the HGT mechanism mediated by *K. pneumoniae* OMVs, specifically how *K. pneumoniae* integrates DNA into OMVs and protects it from extracellular nuclease activity, transferring DNA within vesicles to recipient bacteria. Recipient *K. pneumoniae* acquired and expressed ampicillin resistance after contact with OMVs, demonstrating OMVs’ ability to facilitate intra-species HGT. This mechanism can occur not only within species but also potentially spread between species, further exacerbating the dissemination of resistance genes. Chen et al. (2023) further demonstrated that carbapenem-resistant *K. pneumoniae* (CR-KP) producing KPC-2 carbapenemase can transmit the *blaKPC-2* gene to susceptible strains via OMVs, conferring carbapenem resistance.

OMVs also enhance survival by protecting active resistance proteins. For instance, Hussein et al. (2023) conducted comparative analyses of the OMVs subproteome from polymyxin-sensitive and resistant *K. pneumoniae* isolates, finding that polymyxin-resistant *K. pneumoniae* delivers protein cargo promoting resistance and cell repair processes in response to polymyxin B, including proteins involved in outer membrane remodeling, CAMP resistance, *β*-lactam resistance, and quorum sensing. Other studies have found that OMVs from CRKP are rich in KPC enzymes, and OMVs can protect KPC enzymes from degradation by proteinase K, thereby enhancing CRKP’s meropenem hydrolysis activity (Yao et al., 2023).

The study by Fan et al. (2023) supports this perspective, revealing through mass spectrometry analysis that the OMVs of CRKP contain various proteins associated with antibiotic resistance. Among these, the multidrug-resistant outer membrane protein MdtQ was identified to possess a unique three-dimensional structure and biological pocket, potentially offering a theoretical basis for controlling the spread of bacterial resistance. Another study by their team (Fan et al., 2024) further explored the protein composition of OMVs in CRKP treated with meropenem. The research found that meropenem-induced OMVs are enriched with proteins related to virulence factors, resistance, stress response, and cellular metabolism. These differential proteins primarily participate in metabolic processes, cellular components, catalytic activity, and binding functions, involving chromosome partitioning protein MukB, heat shock protein GroEL, *γ*-glutamyl phosphate reductase, among others. Their protein interaction network indicated that DNA polymerase I and phenylalanyl-tRNA synthetase *β* subunit have the highest connectivity. Parallel reaction monitoring (PRM) validation revealed a significant increase in the abundance of virulence-related proteins such as fimbrial protein and RNA polymerase *σ* factor RpoD. Additionally, OMVs were found to reduce ATP concentration in RAW264.7 cells, suggesting that meropenem-induced OMVs enhance bacterial environmental adaptation and pathogenicity by enriching stress, resistance, and virulence-related proteins.

### 4.2 Escherichia coli

Bielaszewska et al. (2020) showed that OMVs from *E. coli* O104: H4 strains carrying CTX-M-15 can package plasmid-borne *blaCTX-M-15* and *blaTEM-1* genes and transfer them to other Enterobacteriaceae. Transfer frequency increased in simulated intestinal environments and under ciprofloxacin stress, highlighting OMVs as efficient vectors for resistance-gene dissemination.

Kim et al. (2020) and Kim et al. (2018) further elucidated the role of OMVs in *β*-lactam resistance. Their research found that OMVs secreted by resistant *E. coli* significantly upregulate porins such as OmpC and OmpF, as well as β-lactamases like Blc1. These OMVs enhance the survival of susceptible strains in β-lactam antibiotic environments by protecting them from antibiotic exposure. Within the OMV lumen, β-lactamases degrade antibiotics, while porins transport antibiotics into the OMV lumen to assist the β-lactamases.

Li et al. (2024) found that amoxicillin exposure in extended-spectrum β-lactamase (ESBL)-producing *E. coli* increased OMV protein concentration, especially CTX-M-55 ESBLs. The OMV biogenesis protein YdcZ was critical for vesicle formation and protein cargo transport, interacting with YdiH and BssR to promote vesicle secretion and resistance spread.

Additionally, Li et al. (2025) investigated the impact of OMVs from *E. coli* carrying New Delhi metallo-β-lactamase 5 (NDM-5) on resistance. These OMVs significantly reduce the bactericidal effect of meropenem on susceptible *E. coli* and enhance bacterial resistance in a Galleria mellonella infection model. This suggests that OMVs not only function *in vitro* but also promote resistance dissemination during host infection. Similarly, studies found that NDM-1, anchored as a lipoprotein in the outer membrane of *E. coli*, can be selectively secreted into OMVs. OMVs-NDM-1 can protect carbapenem-sensitive bacteria from meropenem killing *in vivo*. In the Galleria mellonella infection model, OMVs-NDM-1 significantly increase the survival rate of susceptible *E. coli* under meropenem treatment, maintaining stable activity for up to 6 h, and providing better protection than free NDM-1 (Martinez et al., 2021). Further research showed that when NDM-1 expressing *E. coli* co-infects with meropenem-sensitive *Pseudomonas aeruginosa*, the released OMVs-NDM-1 can cross-protect *P. aeruginosa*, with the protection efficiency positively correlated with the secretion level of NDM-1 in OMVs (Martinez et al., 2021). This study confirms in vivo that OMVs-NDM-1 mediate cross-species resistance protection through protein transport rather than gene-level transfer.

Regarding polymyxin resistance, Li et al. (2021) examined OMVs from mcr-1–positive *E. coli,* finding that they could protect susceptible strains from polymyxin B. However, protection was weaker than in other mechanisms, likely due to lipid A modification associated with *mcr-1*.

Finally, Ruelens and De Visser (2021) explored adaptive dynamics in a droplet evolution system. In small populations (~27,000 effective size), wild-type *E. coli* under cefotaxime pressure commonly evolved nlpI inactivation—mutations that increase OMV production—conferring moderate resistance. In mutS-deficient strains with a 30-fold elevated mutation rate, nlpI mutations also occurred but were outcompeted by high-benefit mutations such as acrB/acrR efflux pump activation. This suggests that in small populations with lower mutation rates, OMV overproduction becomes a preferred adaptive strategy under low antibiotic pressure, enhancing all OMV-associated resistance processes.

### 4.3 Serratia marcescens

Zhang et al. (2024) discovered that OMVs are crucial vectors for the HGT of the carbapenem resistance gene blaNDM-1. This study provides key experimental evidence showing that *Serratia marcescens* S50079K, a variant strain isolated from the blood of a patient with acute pancreatitis, acquired the *blaNDM-1* gene via OMVs from the *Providencia rettgeri* strain P50213. Researchers confirmed that OMVs can transfer transposable units (TUs) containing the complete *blaNDM-1* gene, facilitating effective interspecies dissemination of resistance genes. Although other transfer mechanisms such as plasmid conjugation were observed, OMVs were identified as an independent mechanism for the transfer of *blaNDM-1* from *Providencia* to *Serratia*. This finding highlights the critical role of OMVs in driving the spread of high-level resistance genes like *blaNDM-1* among different pathogens in clinical settings, such as within patients. The study also noted clonal dissemination in hospital surveillance, where eight carbapenemase-producing *Serratia marcescens* strains evolved over 4 years from carrying only *blaKPC-2* to simultaneously carrying both *blaKPC-2* and *blaNDM-1*. This further underscores the alarming reality of accelerated resistance spread due to gene transfer mechanisms, including OMVs (Zhang et al., 2024).

### 4.4 Acinetobacter baumannii

*Acinetobacter baumannii* is a significant multidrug-resistant pathogen with complex resistance mechanisms. Recent studies have revealed multiple functions of OMVs in the resistance mechanisms of *A. baumannii*, including protecting bacteria from antibiotic attack, spreading resistance genes, and acting as “decoy” strategies to evade antibiotics.

Capodimonte et al. (2025) found that the OXA-type *β*-lactamases OXA-23 and OXA-24/40 in *A. baumannii* are not soluble periplasmic proteins, as traditionally assumed, but instead are anchored as lipoproteins in the outer membrane and can be secreted via OMVs. This membrane-bound property markedly improves the efficiency of packaging these enzymes into OMVs, thereby protecting *A. baumannii* and other susceptible bacteria, such as *Escherichia coli* and *Pseudomonas aeruginosa*, from β-lactam antibiotics. Additionally, Monteiro et al. (2025) confirmed that carbapenem-resistant *A. baumannii* spreads carbapenemases like NDM-1 and OXA-97 via OMVs, which can hydrolyze imipenem and protect susceptible strains from carbapenem antibiotics.

Another study revealed the protective mechanism of OMVs under antibiotic stress. It was found that multidrug-resistant *A. baumannii* exposed to the novel synthetic fluorocycline antibiotic eravacycline showed significant upregulation of drug efflux pumps (RND and MFS families), ribosomal proteins, and stress-related genes in the bacterial cells, while the OMV proteome was enriched with ribosomal proteins, outer membrane proteins OmpA and Omp38, chaperone proteins, and resistance-related proteins (such as β-lactamases). Transmission electron microscopy revealed increased OMV production in the antibiotic-induced group, and LC–MS/MS analysis showed a higher proportion of stress- and survival-related proteins in OMVs. These findings suggest that under antibiotic pressure, OMVs may act as a “backup defense system,” selectively concentrating stress proteins to enhance bacterial survival (Kesavan et al., 2020).

Kim et al. (2021) discovered that the zinc uptake regulator in *A. baumannii* regulates lipoprotein A (ZrlA), which influences peptidoglycan dynamics and participates in bacterial morphogenesis and OMV generation. ZrlA exhibits D-alanyl-D-alanine carboxypeptidase activity, and its deletion led to a 9.7-fold increase in OMV production in the *A. baumannii* ATCC 17978 mutant strain ΔzrlA compared to the wild type, with reduced OMV particle size but no significant difference in protein profiles. Furthermore, the ΔzrlA mutant showed increased sensitivity to antibiotics like gentamicin, reduced morphological heterogeneity in the stationary phase, and significantly increased cytotoxicity of OMVs to A549 cells. This study revealed that ZrlA negatively regulates OMV generation by maintaining the cross-linking between peptidoglycan and the outer membrane, and its absence may promote OMVs release and enhance virulence by disrupting membrane integrity.

Park et al. (2021) demonstrated that *A. baumannii* employs an OMV-mediated “decoy” strategy to resist polymyxins. In their study, polymyxin B-resistant *A. baumannii* strains showed upregulation of the pmr operon and reduced expression of membrane-associated proteins (OmpA, OmpW, BamE), leading to excessive OMV production and enhanced biofilm formation. They used lipid-selective dyes (FM4-64) and dansyl-PMB to visualize the binding of PMB to purified OMVs stained with PMRHigh. The results showed overlapping images, clearly demonstrating direct binding of PMB to OMVs. *In vitro* anaerobic human gut microbiota analysis indicated that OMVs could completely protect microbial communities from the effects of polymyxin B. In a wax moth larva infection model, OMVs increased larval mortality by protecting *A. baumannii* from polymyxin B.

### 4.5 Pseudomonas aeruginosa

*Pseudomonas aeruginosa* is a significant multidrug-resistant pathogen with complex resistance mechanisms. Recent studies have revealed multiple roles of OMVs in the resistance mechanisms of *P. aeruginosa*, including enhancing resistance to polymyxins, regulating biofilm formation and disassembly, promoting horizontal gene transfer, and protecting bacteria from carbapenem antibiotics.

Gram-negative bacteria display remarkable complexity and adaptability in establishing and maintaining biofilm systems. Biofilms are multicellular aggregates formed by bacteria on surfaces, encased in a self-produced extracellular matrix composed mainly of polysaccharides, proteins, and nucleic acids (Costerton et al., 1999). The resistance of Gram-negative bacterial biofilms mainly stems from several factors. Firstly, the biofilm matrix can block or slow down antibiotic penetration, making it difficult for drugs to reach deep-seated bacteria. Secondly, the metabolic activity of bacteria within biofilms is reduced, decreasing antibiotic targets (Nahum et al., 2025). Moreover, the biofilm environment promotes horizontal gene transfer, increasing the spread of resistance genes within the bacterial community. OMVs play a crucial role in the biofilm dispersion process of *P. aeruginosa*. Studies have found that the quorum sensing signal molecule Pseudomonas quinolone signal (PQS) can induce OMV production, significantly enhancing this process during biofilm dispersion. Biofilm dispersion ability is notably impaired in PQS biosynthesis mutants and receptor mutants, while exogenous PQS addition or genetic complementation can restore this function. Further research indicates that purified OMVs possess enzymatic activity to degrade extracellular proteins, lipids, and DNA, suggesting that PQS-induced OMVs may facilitate bacterial escape by coordinating the degradation of biofilm matrix components (Cooke et al., 2020). Esoda and Kuehn (2019) further demonstrated that leucine aminopeptidase (PaAP) secreted by *P. aeruginosa* substantially influences the composition and structure of early biofilms by modulating the antimicrobial biofilm activity of OMVs. Loss of PaAP results in denser biofilms with reduced matrix polysaccharide content, whereas OMVs from PaAP-producing strains can reshape biofilm matrix and colony structure through protease-mediated detachment. Notably, this OMV-mediated antimicrobial biofilm activity also affects biofilms of other *P. aeruginosa* strains and *K. pneumoniae*. Saad et al. (2024) reported that OMVs released by *P. aeruginosa* PAO1 biofilms at different growth stages exhibit dual effects on biofilm dynamics. OMVs from biofilms in the logarithmic growth phase (G-OMVs) markedly promote biofilm development, whereas OMVs from the death/survival phase (D-OMVs) strongly inhibit biofilm formation and growth. Proteomic analysis revealed that D-OMVs carry proteins involved in growth inhibition, reactive oxygen species (ROS) production, and iron acquisition—processes that promote survival or induce cell death. Moreover, the inhibitory effect of D-OMVs on mature biofilms is amplified in the presence of iron ions, potentially implicating iron-dependent ferroptosis pathways. These results underscore the pivotal role of *P. aeruginosa* OMVs throughout the biofilm life cycle.

Johnston et al. (2023) focused on the role of *P. aeruginosa* OMVs in horizontal gene transfer under different growth conditions. They found that OMVs produced during planktonic growth can encapsulate and protect plasmid DNA from DNase degradation and transfer antibiotic resistance genes to recipient *P. aeruginosa* more efficiently than using plasmid DNA alone. Biofilm-derived OMVs, although smaller in size, carry more plasmid DNA and have a significantly higher efficiency in transforming recipient bacteria compared to planktonic-derived OMVs, indicating the important influence of bacterial growth conditions on the DNA packaging and HGT capability of *P. aeruginosa* OMVs.

Zhang et al. (2023) discovered that OMVs from *K. pneumoniae* carbapenemase-producing strains can protect *P. aeruginosa* from imipenem’s bactericidal effects. These OMVs protect *P. aeruginosa* by hydrolyzing antibiotics in a dose- and time-dependent manner and may induce resistance mutations.

Additionally, studies have found that *P. aeruginosa* OMVs can enhance resistance to polymyxin B, and both OMVs and sublethal concentrations of polymyxin B can inhibit the transcription levels of genes related to the quorum sensing (QS) system. Their synergistic effect further inhibits the QS system of *P. aeruginosa*, resulting in reduced virulence factor secretion, impaired bacterial motility, and significantly decreased biofilm formation ability (Chen et al., 2024).

### 4.6 Elizabethkingia anophelis

Chiang et al. (2022) discovered that the OMVs released by pandrug-resistant *Elizabethkingia anophelis* under antibiotic pressure exhibit unique physical properties and proteomic characteristics. Using nanoparticle tracking analysis, transmission electron microscopy, and proteomics analysis, they found that different antibiotic treatments affect the size, concentration, and surface charge of OMVs, with imipenem-induced OMVs (iOMVs) showing the highest particle concentration and better uniformity. The proteins within OMVs are primarily involved in processes related to metabolism, survival, defense, and antibiotic resistance, including Rag/Sus family proteins, molecular chaperone GroEL, isoprenyl transferase, and HmuY family proteins. Protein interaction network analysis revealed that iOMVs are significantly enriched in pathways related to cell membrane structure, adenosine binding, serine-type peptidase activity, glycosyl compound metabolism, and cation binding.

Furthermore, *in vitro* experiments demonstrated that iOMVs can enhance bacterial immune evasion and cytotoxicity, although no significant carbapenemase activity was detected. This suggests that the role of OMVs in bacterial response to antibiotic pressure is more inclined toward promoting survival rather than directly mediating resistance (Chiang et al., 2022).

### 4.7 Aeromonas hydrophila

Lin et al. (2022) extracted and characterized OMVs from oxytetracycline-sensitive (OXY-S) and oxytetracycline-resistant (OXY-R) *Aeromonas hydrophila* strains, revealing pronounced differences in the protein profiles between OMVs-S and OMVs-R. In LB medium without antibiotics, neither OMVs-S nor OMVs-R significantly affected the growth of OXY-S strains. However, under oxytetracycline treatment, both types of OMVs markedly enhanced bacterial resistance, with OMVs-R exerting a more pronounced protective effect. Quantitative proteomic analysis using data-independent acquisition (DIA) identified 626 OMV-associated proteins, including 12 differentially expressed proteins—seven upregulated and five downregulated. Functional experiments demonstrated that deletion of iscS, icd, and rpsF genes significantly reduced bacterial resistance to oxytetracycline. Moreover, recombinant expression of upregulated proteins, followed by crude OMV extraction, revealed that certain recombinant protein-enriched OMVs substantially increased bacterial survival under antibiotic pressure. Notably, recombinant RpsF (rRpsF) was capable of directly binding oxytetracycline (Lin et al., 2022).

Seike et al. (2020) showed in vitro that most *A. hydrophila* strains produce biofilms composed of polysaccharides containing N-acetylglucosamine, extracellular nucleic acids, and proteins, some of which share homology with outer membrane proteins of other Gram-negative bacteria. The addition of *A. hydrophila* OMVs promoted biofilm formation in a dose-dependent manner. Treatment of OMVs with proteinase K markedly reduced this effect, indicating that OMV-associated proteins play a critical role in biofilm development.

Taken together, these findings suggest that upregulated metabolic proteins in OMVs from resistant strains may enhance energy supply for biofilm matrix synthesis, while proteins such as rRpsF that directly bind antibiotics can lower the concentration of free drugs within the biofilm. This combination forms a synergistic “physical barrier–molecular trap” defense, illustrating that OMV-mediated bacterial resistance can have multifaceted outcomes.

### 4.8 Neisseria gonorrhoeae

Dhital et al. (2022) discovered that resistant *Neisseria gonorrhoeae* can secrete *β*-lactamase via OMVs, which protect otherwise sensitive strains from β-lactam antibiotics like amoxicillin. The β-lactamase in OMVs is enzymatically active and can transfer the enzyme to the periplasmic space of sensitive bacteria through membrane fusion, thereby increasing their minimum inhibitory concentration (MIC). This protective effect is not limited to the same species and is also effective against sensitive *E. coli*. The study also found that the protection provided by OMVs to sensitive strains is time-dependent, significantly increasing MIC initially but gradually weakening over time.

### 4.9 Helicobacter pylori

The treatment of *Helicobacter pylori* infection typically involves amoxicillin combined with clarithromycin or metronidazole along with proton pump inhibitors, but the failure rate of first-line triple therapy has been increasing, especially those containing clarithromycin (Duan et al., 2025). Murray et al. (2020) found that OMVs produced by *H. pylori* can enhance bacterial survival under stress and antibiotic exposure through various mechanisms. Specifically, OMVs protect *H. pylori* from hydrogen peroxide *in vitro* in a dose-dependent manner and show some protective effects against clarithromycin and levofloxacin, but are ineffective against ampicillin and metronidazole. Additionally, OMVs enable *H. pylori* to grow in the presence of the antimicrobial peptide LL-37, suggesting that OMVs might interact with LL-37 to release nutrients necessary for bacterial growth.

**Figure 1:**
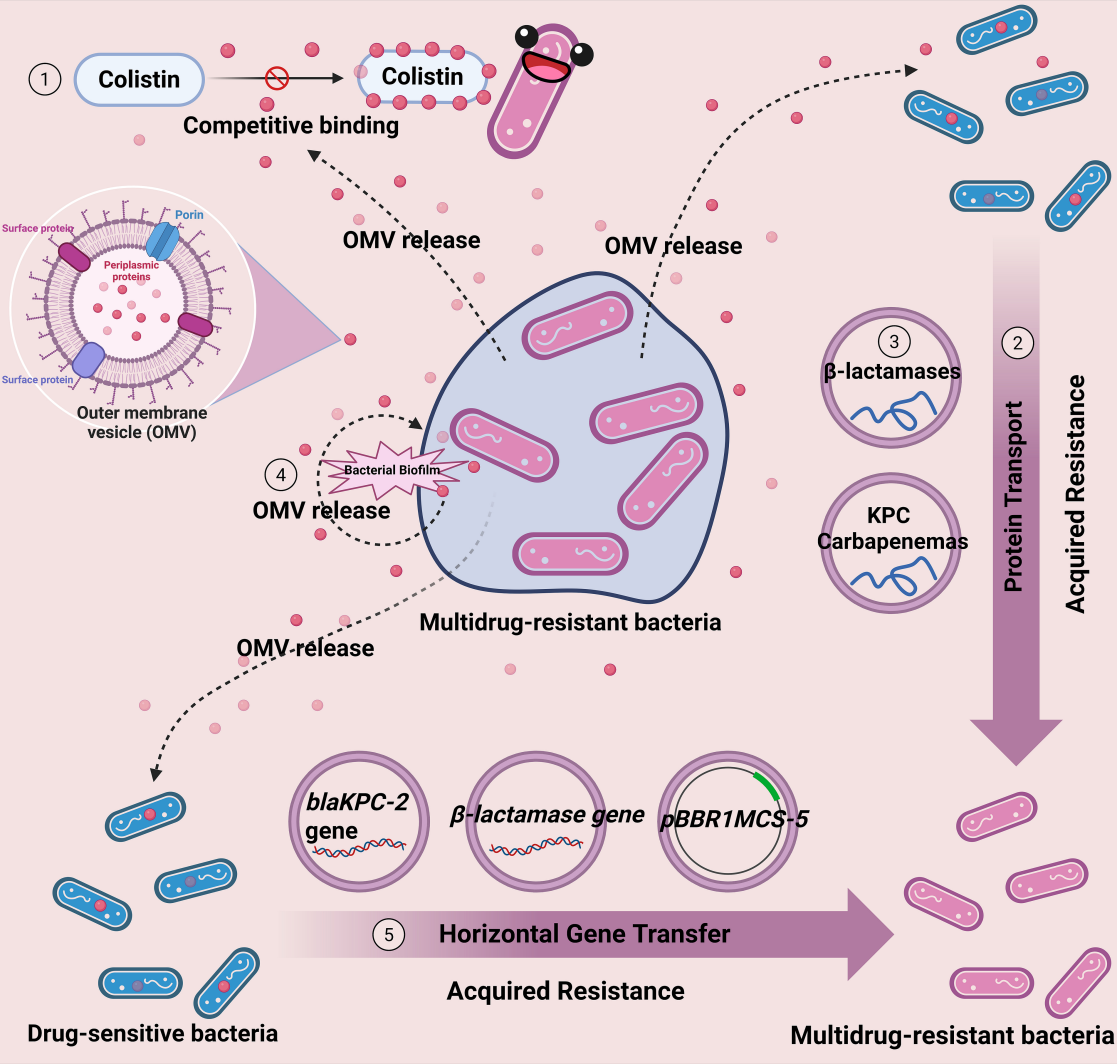
Mechanisms of OMV-mediated antibiotic resistance in bacteria. OMVs from pathogenic bacteria contribute to antibiotic resistance by ① acting as decoys to bind and neutralize antibiotics, ② transferring resistance genes and proteins to other bacteria, ③ protecting key enzymes (e.g., β-lactamases) from degradation, ④ facilitating biofilm formation and maintenance, and ⑤ promoting horizontal gene transfer within and between species.

Based on the comprehensive analysis in Sections 4.1 to 4.9, bacterial OMVs play multiple roles in bacterial resistance mechanisms. OMVs can directly protect bacteria from antibiotic attacks and enhance bacterial resistance through various mechanisms, including transferring resistance genes, preserving the activity of key enzymes, and promoting outer membrane remodeling (Figure 1). Furthermore, OMV-mediated horizontal gene transfer exacerbates the spread of resistance genes, making the issue of antibiotic resistance even more complex.

## 5 Clinical applications of bacterial outer membrane vesicles in anti-infective therapy

### 5.1 Reducing OMV secretion to treat/delay multidrug resistance

As discussed in preceding sections, bacterial OMVs play a pivotal role in the dissemination of antimicrobial resistance. OMVs can confer direct or indirect protection against antimicrobials through competitive drug binding, horizontal gene transfer, and delivery of resistance-related proteins. Consequently, strategies aimed at reducing OMV secretion may represent an effective approach to delay the development of multidrug resistance.

Kosgodage et al. (2019) demonstrated that inhibition of peptidylarginine deiminase (PAD) activity using PAD inhibitors markedly reduces OMV release and enhances bacterial susceptibility to a range of antibiotics. PAD was identified as a critical regulatory factor in OMV secretion in both Gram-negative and Gram-positive bacteria. By attenuating OMV release, PAD inhibitors diminished bacterial resistance and, importantly, decreased the minimum inhibitory concentrations (MICs) of certain antibiotics. This allows for lower therapeutic doses, potentially minimizing cytotoxic effects on host cells.

Grande et al. (2021) discovered that natural compounds carvacrol and thymol can selectively inhibit the carbonic anhydrase enzymes (HpCAα and HpCAβ) of *H. pylori*, significantly weakening biofilm formation and OMV release at sub-minimum inhibitory concentrations. This reduction in extracellular DNA (eDNA)-mediated resistance spread further contributes to resistance management. Moreover, carvacrol and thymol exhibit low toxicity to AGS cells, ensuring safe administration through oral routes and upon contact with the gastric environment.

### 5.2 Using OMVs as adjuvants in antibacterial vaccines

OMVs, as natural immunogens, hold great potential for development as antibacterial vaccines. Balhuizen et al. (2022) demonstrated that OMVs are viable vaccine candidates against Gram-negative bacteria, with their immunogenicity modulated by host defense peptides (HDPs) such as LL-37, CATH-2, PMAP-36, and K9CATH. The modulatory effects varied depending on the OMV type—whether produced spontaneously or induced by heat. OMVs activated multiple Toll-like receptors (TLRs), including TLR2, TLR4, TLR5, and TLR9, with TLR4 activation being predominant. These results suggest that OMVs can activate macrophages, and that cathelicidins modulate immune responses via interactions with OMVs, with modulation dependent on OMV induction method and host species.

Baker et al. (2024) used the Kymouse platform to immunize mice with OMVs and screened for protective monoclonal antibodies (mAbs) against carbapenem-resistant *A. baumannii*. Among them, mAb1416 targets KL49 capsular polysaccharide, preventing infections related to neonatal sepsis strains in Asia. Ahmad et al. (2019) observed that clinical *A. baumannii* isolates can reversibly transition between opaque and translucent colonies. These transitions were associated with phenotypic changes in morphology, surface motility, biofilm formation, antibiotic resistance, and virulence. The translucent phenotype formed denser biofilms, produced more pili, and secreted increased quantities of OMVs, while reducing the fertility of *Caenorhabditis elegans*. In RAW 264.7 macrophages, OMVs from opaque colonies were more immunogenic than those from translucent colonies, suggesting the potential utility of opaque-colony OMVs in novel immunotherapeutic strategies against *A. baumannii* infections.

OMVs can not only serve as mediators for resistance spread but also enhance bacterial sensitivity to antimicrobial agents through specific immune strategies. Huang et al. (2019) showed that antisera generated by immunizing mice with OMVs from highly resistant *A. baumannii* significantly increased bacterial susceptibility to multiple antibiotics. *In vitro*, combining anti-OMV sera with quinolone antibiotics substantially enhanced bacterial sensitivity. In murine sepsis and pneumonia models, both active and passive immunization with these antibodies improved survival, reduced organ bacterial load, and increased quinolone susceptibility. Protein target analysis identified several porins, suggesting that anti-OMV antibodies enhance antibiotic accumulation by modulating porin function. Building on this work, the same team (Huang et al., 2020) developed drug-loaded OMVs based on OMV-mediated efflux mechanisms, enabling effective drug delivery into pathogenic bacteria. In a murine intestinal infection model, low-dose oral administration prolonged drug retention in the intestine, markedly reduced bacterial loads in intestinal and fecal samples, and exhibited good biocompatibility—providing a dual “sensitization–delivery” strategy to combat resistance.

Jones et al. (2024) modified OMVs by replacing the PorB of *Neisseria gonorrhoeae* with that of *Neisseria meningitidis* and knocking out the RmpM gene. Compared to wild-type OMVs, the modified OMVs induced higher levels of IgG antibodies in mouse models, with a more diverse antibody response. Additionally, the modified OMVs induced a Th1-biased immune response, characterized by increased IgG2a antibody levels and IFN-*γ* production in spleen cells.

Pritsch et al. (2021) selected *E. coli* CFT073 and three Multidrug-Resistant Gram-Negative Organisms (MDRGNO) strains that had caused severe human infections, administering their OMVs intranasally to mice. The results showed that OMVs could induce specific IgM and IgG antibodies against the corresponding MDRGNO in mice, comparable to antibody responses in sera from patients previously infected with the respective bacteria. Furthermore, mice receiving intranasal OMV administration showed no local or systemic toxic reactions, indicating good vaccine tolerance.

For *H. pylori*, Song et al. (2020) combined OMVs from strain 7.13 with two vaccine types—outer membrane proteins (OMP) and whole-cell vaccine (WCV)—to immunize mice. OMVs used as adjuvants significantly increased anti-OMP IgG titers, enhanced gastric mucosal immunity, promoted Th1/Th2/Th17 responses with a Th2/Th17 bias, and effectively reduced gastric *H. pylori* colonization. Compared with cholera toxin (CT) as an adjuvant, OMVs provided superior immune protection enhancement. Liu et al. (2019) systematically analyzed the protein composition of OMVs derived from *H. pylori* strain 7.13 adapted to guinea pigs, finding them rich in outer membrane, periplasmic, and extracellular proteins. In C57BL/6 mouse models, OMVs induced stronger humoral and significantly higher mucosal immune responses compared to *H. pylori* whole-cell vaccines using CT as an adjuvant. OMVs primarily induced a Th2-biased immune response, significantly reducing bacterial load following infection with *H. pylori* Sydney strain 1. Maiti et al. (2021) developed a bivalent non-typhoidal Salmonella OMVs vaccine that reduced Salmonella-induced gastrointestinal infections through passive immunization in mouse models, demonstrating broad-spectrum protective potential.

### 5.3 Anti-biofilm activity

Gurunathan et al. (2023) reported that outer membrane vesicles derived from *P. aeruginosa* (PAOMVs) exhibit potent antibacterial and anti-biofilm activities against *Streptococcus mutans*. The study demonstrated that PAOMVs inhibit both cellular viability and biofilm formation of *S. mutans* in a dose-dependent manner. This inhibitory effect is mediated by excessive generation of reactive oxygen species and concomitant reductions in antioxidant markers such as glutathione, superoxide dismutase, and catalase, leading to lipid peroxidation and impaired cellular metabolic activity. Furthermore, PAOMVs significantly suppress lactate dehydrogenase activity and ATP production, while promoting leakage of intracellular proteins and sugars, ultimately enhancing bacterial cell death.

Notably, when PAOMVs were combined with sub-lethal concentrations of antibiotics, they further amplified cytotoxicity and biofilm inhibition against *S. mutans*. This suggests the potential application of PAOMVs as adjuvants to enhance the efficacy of sub-lethal antibiotic doses in bacterial infection management. However, the underlying molecular mechanisms remain unclear, and the dual role of *P. aeruginosa* OMVs—given their capacity to mediate horizontal transfer of antibiotic resistance genes (Johnston et al., 2023) —presents safety concerns. Future studies are therefore expected to focus on elucidating these mechanisms to enable the safe and consistent exploitation of their antibacterial properties.

### 5.4 Engineered OMVs

The development of engineered OMVs represents a promising new strategy in antimicrobial therapy (Huang et al., 2016). By modifying OMVs through genetic engineering, membrane surface functionalization, or targeted cargo loading, researchers have transformed these natural nanocarriers into versatile platforms for drug delivery and vaccine development (Tang et al., 2024). Engineered OMVs can be tailored to enhance immune responses, deliver antimicrobial agents more efficiently, and reduce inflammation or off-target effects (Chen et al., 2010). These advances not only help overcome the challenges of antibiotic resistance but also open up new possibilities for precise and effective treatment of bacterial infections.

Park et al. (2023) developed synthetic bacterial vesicles (SyBVs) using engineered vesicle technology, which markedly reduced macrophage- and murine inflammation while eliciting antigen-specific adaptive immunity comparable to that of natural OMVs. SyBVs derived from different bacterial sources exhibited distinct effects; for example, *P. aeruginosa* SyBVs conferred protection against bacterial challenge in mice, attenuating lung cell infiltration and inflammatory cytokine production, while *E. coli* SyBVs protected mice from *E. coli* sepsis. Moreover, SyBVs were engineered to display the SARS-CoV-2 S1 protein on their surface, inducing specific anti-S1 antibodies and T-cell responses.

Singh et al. (2024) created outer membrane hybrid vesicles (OM-Hybrids) by incorporating synthetic lipids into OMVs via rapid freeze–thaw cycles. These were used to construct planar outer membrane-supported bilayers (OM-SBs) that mimic natural outer membrane–antimicrobial peptide interactions, providing a biomimetic framework for antimicrobial delivery system design. Separately, OMVs incorporating bovine serum albumin nanoparticles via hydrophobic interactions produced uniform (~100 nm), structurally stable vaccines, which significantly elevated CRKP-specific antibody titers and improved murine survival after lethal CRKP challenge (Wu et al., 2020).

To overcome acquired bacterial resistance due to porin protein loss, Wu et al. (2024) studied a novel antibiotic delivery platform synthesized using OMVs. They systematically evaluated the efficiency of passive and active loading methods (such as electroporation and ultrasonic treatment) *in vitro* and *in vivo*, finding that low-voltage electroporation (200 and 400 V) achieved the highest drug encapsulation efficiency. The study also demonstrated that imipenem delivered via OMVs significantly enhanced efficacy against *E. coli*.

Liu et al. (2024) discovered that genetically engineered OMVs from *Salmonella Typhimurium* serve as antigen delivery platforms, presenting key antigens UreB, CagA, and VacA from *H. pylori* on OMV surfaces via the hemoglobin protease (Hbp) autotransport system, effectively inducing protective immunity against *H. pylori*. The study first screened for optimal *Salmonella* mutant strains ΔrfbP ΔfliC ΔfljB ΔompA, whose secreted OMVs significantly increased IgG levels in mouse serum and IgA levels in gastric mucosa, inducing a Th1 and Th17-biased cellular immune response. Further antigen combination optimization revealed that OMVs co-delivering UreB and CagA exhibited the best protective effect in mouse models, significantly reducing urease activity and bacterial load post- *H. pylori* infection while stimulating antigen-specific T-cell responses.

Research on engineered extracellular vesicles (EVs) targeting Gram-positive pathogens is also progressing. For example, Yang et al.’s (2025) research reveals a novel application of bacterial EVs in the targeted delivery of antibiotics to treat infections caused by both intracellular and extracellular *Staphylococcus aureus*. The team developed a pathogen-targeting biomineralized EVs system, where EVs derived from *E. coli* were modified with a hydroxamate-type siderophore to specifically target intracellular *Staphylococcus aureus*. The EVs surface was further coated with pH-sensitive calcium carbonate (CaCO3) to enable targeted drug release in the infection microenvironment. The EVs were loaded with the antimicrobial drugs lysostaphin and mupirocin. This biomineralized EVs system demonstrated effective eradication of both extracellular and intracellular *S. aureus in vitro* and *in vivo*, showing superior antibacterial efficacy compared to free antibiotics or unmodified EVs. Gao et al. (2019) discovered that nanoparticles (NP@EV) wrapped with EVs membranes secreted by *S. aureus* serve as active targeting drug carriers, efficiently targeting and eliminating *S. aureus* within macrophages. Compared to nanoparticles wrapped with polyethylene glycol lipid bilayers (NP@Lipo) or *E. coli* OMV membranes (NP@OMV), NP@EV exhibited stronger targeting ability and therapeutic effects both in vitro and in vivo, particularly demonstrating significant antibacterial effects in kidneys and lungs where the infection burden is highest. Chen et al. (2025) found that biomimetic nanoparticles constructed by coating mesoporous silica nanoparticles (MSNs) with EVs efficiently deliver peptide nucleic acids (PNAs) to *S. aureus*. This nanostructure, with EVs as the shell and PNA-loaded MSN as the core, selectively enhances bacterial uptake of nanoparticles through EV targeting, showing significantly superior antibacterial activity compared to free PNA and PNA@MSN without EV coating.

### 5.5 Probiotic-derived OMVs in adjunctive treatment of bacterial infections

Hu et al. (2020) discovered that OMVs derived from the probiotic *E. coli* Nissle 1917 (EcN-OMVs) can be internalized by RAW264.7 macrophages. At moderate concentrations, EcN-OMVs promote macrophage proliferation, enhance the activity of immune-related enzymes such as acid phosphatase and inducible nitric oxide synthase, and improve phagocytic function. They also induce higher secretion levels of the anti-inflammatory cytokine IL-10 compared to pro-inflammatory factors IL-6 and TNF-*α*, while regulating the production of Th1-type cytokine IL-12 and Th2-type cytokine IL-4, effectively boosting macrophage antibacterial activity against *E. coli*, *Salmonella*, and *S. aureus*.

Similarly, Ma et al. (2025) demonstrated that EcN-OMVs are internalized by RAW 264.7 macrophages and promote M1 polarization via activation of the HIF-1α, mTORC1, and NF-κB signaling pathways, accompanied by metabolic reprogramming. This included enhanced glycolysis and suppression of the tricarboxylic acid (TCA) cycle, elevated intracellular reactive oxygen species (ROS), TNF-α, IL-6, IL-1β, nitric oxide (NO), and ATP levels, as well as increased macrophage proliferation, migration, invasion, and phagocytosis. Metabolomic analysis indicated that metabolites such as stearic acid, branched-chain amino acids, and succinic acid contained within EcN-OMVs act as drivers of this metabolic remodeling and polarization process.

**Figure 2:**
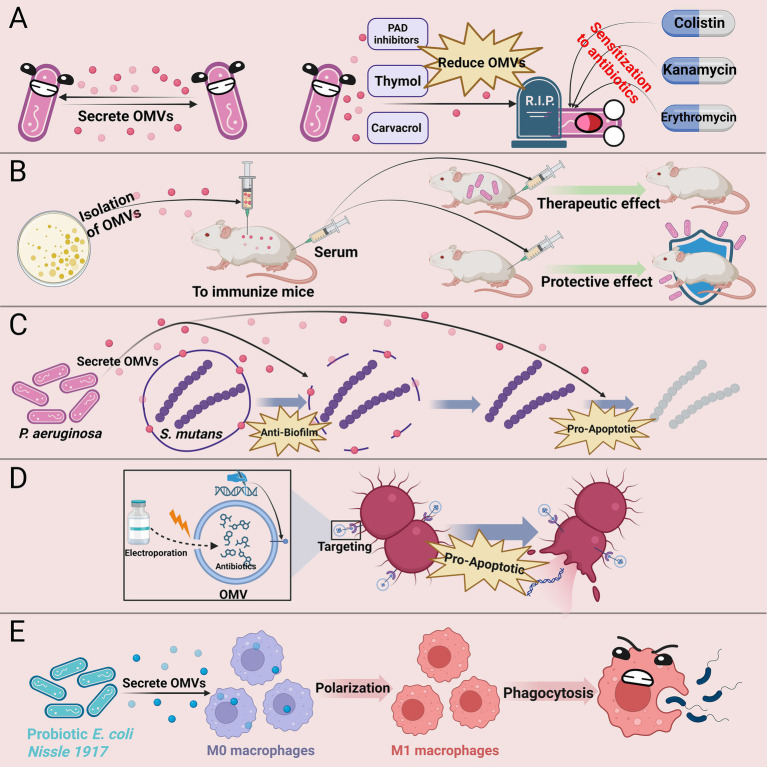
Clinical anti-infective strategies based on bacterial OMVs. **(A)** Inhibiting OMV secretion (e.g., with PAD inhibitors, thymol, or carvacrol) enhances bacterial susceptibility to antibiotics; **(B)** OMV-based vaccines elicit protective immune responses against bacterial infections; **(C)** OMVs from certain bacteria can inhibit biofilm formation by other bacteria and induce apoptosis; **(D)** Engineered OMVs facilitate targeted antibiotic delivery and enhance antibacterial efficacy; **(E)** Probiotic-derived OMVs modulate macrophage function and boost innate immunity. PAD, peptidylarginine deiminase.

In summary, research on the application of bacterial OMVs in clinical anti-infective therapy has made significant progress. Strategies such as reducing OMV secretion, enhancing bacterial sensitivity to antimicrobial agents, using OMVs as adjuvants in antimicrobial vaccines, leveraging the anti-biofilm properties of OMVs, developing engineered OMVs, and utilizing probiotic-derived OMVs in adjunctive treatment offer new approaches to tackle bacterial resistance challenges (Figure 2). The current priorities are: firstly, to thoroughly investigate the specific mechanisms by which OMVs exert antibacterial effects; secondly, to analyze the mechanisms by which engineered OMVs developed from different bacteria exhibit distinct functional roles, thereby enhancing their targeted therapeutic capabilities; additionally, to optimize the construction of engineered OMVs to improve drug loading efficiency and *in vivo* transport capacity; and finally, most critically, to optimize existing OMVs extraction methods to achieve high yield and low cost while preserving the biological characteristics of OMVs as much as possible.

## 6 Conclusion

OMVs, as nanoscale membrane structures secreted by Gram-negative bacteria, play critical roles in bacterial antibiotic resistance and hold significant potential for clinical anti-infective therapy. This review highlights their multifaceted functions in resistance mechanisms, including gene transfer, signal transduction, and biofilm formation, as well as their emerging applications in vaccine development, drug delivery, and immune modulation. OMVs not only serve as tools for bacterial adaptation to environmental pressures but also facilitate the dissemination of resistance genes and bacterial survival strategies.

The biogenesis and functional mechanisms of OMVs are complex, influenced by bacterial species, environmental conditions, and external pressures. Understanding these mechanisms is essential for elucidating their roles in bacterial physiology and host-pathogen interactions. OMVs carry diverse biomolecules, such as proteins, lipids, and nucleic acids, which impact host immune responses and bacterial pathogenicity. Despite their potential, the application of OMVs, particularly engineered OMVs, remains in its early stages, with future research needed to improve their targeting, drug loading efficiency, and in vivo stability.

Future efforts should focus on: (1) elucidating the biogenesis and functional mechanisms of OMVs; (2) developing standardized OMV isolation and purification technologies; (3) optimizing engineered OMVs for targeted therapies; (4) exploring their applications in combating multi-drug-resistant bacterial infections; and (5) investigating their roles in host immune modulation. Interdisciplinary collaboration, integrating advances in molecular biology, nanotechnology, and pharmaceutical chemistry, will be key to unlocking the full potential of OMVs.

In conclusion, as key mediators of bacterial-environment interactions, OMVs provide new perspectives for elucidating bacterial resistance mechanisms and offer important theoretical and technical support for developing novel anti-infective strategies based on “vesicle-to-vesicle” approaches. OMVs are poised to become vital tools in addressing bacterial resistance challenges, paving new paths for global anti-infective therapy. With a deeper understanding of OMV functions and continuous technological advancements, the clinical application potential of OMVs will be further realized, providing stronger guarantees for human health. Through ongoing research and innovation, OMVs will not only achieve breakthroughs in basic science but also play significant roles in clinical applications, ushering anti-infective therapy into a new era.
